# Identifying Vulnerable Brain Networks in Mouse Models of Genetic Risk Factors for Late Onset Alzheimer’s Disease

**DOI:** 10.3389/fninf.2019.00072

**Published:** 2019-12-10

**Authors:** Alexandra Badea, Wenlin Wu, Jordan Shuff, Michele Wang, Robert J. Anderson, Yi Qi, G. Allan Johnson, Joan G. Wilson, Serge Koudoro, Eleftherios Garyfallidis, Carol A. Colton, David B. Dunson

**Affiliations:** ^1^Department of Radiology, Duke University, Durham, NC, United States; ^2^Department of Neurology, Duke University School of Medicine, Durham, NC, United States; ^3^Brain Imaging and Analysis Center, Duke University, Durham, NC, United States; ^4^Pratt School of Engineering, Duke University, Durham, NC, United States; ^5^Department of Biomedical Engineering, University of Delaware, Newark, NJ, United States; ^6^Department of Psychology and Neuroscience, Trinity College of Arts & Sciences, Duke University, Durham, NC, United States; ^7^School of Informatics, Computing, and Engineering, Indiana University Bloomington, Bloomington, IN, United States; ^8^Department of Statistical Science, Trinity College of Arts & Sciences, Duke University, Durham, NC, United States

**Keywords:** mouse model, Alzheimer’s disease, neurodegeneration, magnetic resonance imaging, tractography, tract based analysis, morphometric, diffusion tensor (DT) MRI

## Abstract

The major genetic risk for late onset Alzheimer’s disease has been associated with the presence of APOE4 alleles. However, the impact of different APOE alleles on the brain aging trajectory, and how they interact with the brain local environment in a sex specific manner is not entirely clear. We sought to identify vulnerable brain circuits in novel mouse models with homozygous targeted replacement of the mouse ApoE gene with either human APOE3 or APOE4 gene alleles. These genes are expressed in mice that also model the human immune response to age and disease-associated challenges by expressing the human NOS2 gene in place of the mouse mNos2 gene. These mice had impaired learning and memory when assessed with the Morris water maze (MWM) and novel object recognition (NOR) tests. *Ex vivo* MRI-DTI analyses revealed global and local atrophy, and areas of reduced fractional anisotropy (FA). Using tensor network principal component analyses for structural connectomes, we inferred the pairwise connections which best separate APOE4 from APOE3 carriers. These involved primarily interhemispheric connections among regions of olfactory areas, the hippocampus, and the cerebellum. Our results also suggest that pairwise connections may be subdivided and clustered spatially to reveal local changes on a finer scale. These analyses revealed not just genotype, but also sex specific differences. Identifying vulnerable networks may provide targets for interventions, and a means to stratify patients.

## Introduction

The multifactorial nature of Alzheimer’s disease AD has led to multiple hypotheses for disease onset and progression (Devanand et al., 2007), yet its etiology is not known. While pathological biomarkers have been well defined, cross-disciplinary approaches are critical to integrate knowledge on the spatiotemporal evolution of AD. Additionally, sensitive tools that permit early detection and monitoring changes are critical to enable useful interventions. Analyses of Aβ plaques and tau tangles are considered to provide the “classical” biomarkers of AD. But Aβ plaques and tau tangles are accompanied by neuronal dystrophy and loss (Serrano-Pozo et al., 2011). For the small percentage of individuals with the mutated forms of these proteins, the onset and progression of these biomarkers are clearly dependent on the genetic mutations. However, most individuals afflicted with AD have a late onset form of AD (LOAD). There is a long asymptomatic period that often precedes the overt phases, and during this time other processes besides those centered directly on Aβ plaque formation may be activated to cause neurodegenerative diseases. These processes may involve microglia, astrocytes, and the vasculature (De Strooper and Karran, 2016). In particular, for LOAD, there is a pressing need to better understand the role of non-classical risk factors in AD including age, sex, and genes, and how they interact to modulate the brain response to stressors (Sala Frigerio et al., 2019).

One of the best known genetic risk factors for LOAD is conferred by the APOE4 genotype (Schellenberg, 1995; Huynh et al., 2017). The APOE4/4 genotype is associated with a 30–55% risk of developing mild cognitive impairment (MCI) or AD by age 85, compared to a 10–15% risk for the APOE 3/3 genotype. Still, the precise cause for increased risk, or resilience conferred by the different APOE alleles, and the mechanisms mediating these relationships are poorly understood. While these risk factors may influence the brain levels of Aβ and hyperphosphorylated tau, it is likely that their underlying mechanisms contributing to AD onset, progression and overall pathology will vary. Besides being recognized as a major genetic risk for AD, the presence of APOE4 has been linked to other neurodegenerative diseases. These include age related macular degeneration, age related hearing loss, dementia with Lewy bodies and Parkinson’s disease. APOE4 provides increased susceptibility to neuromuscular conditions including diabetic neuropathy and immunodeficiency viral neuropathy (Bedlack et al., 2000; Pankratz et al., 2006). Moreover, APOE4 is as a risk factor for cardiovascular disease, and stroke (Tudorache et al., 2017; Femminella et al., 2018; Belloy et al., 2019). Due to its complex, not yet completely understood role, we have examined in this work primarily phenotypes relevant to AD.

MRI can provide such phenotypes, e.g., early regional atrophy (Jack et al., 1999), and quantitative biomarkers that can be analyzed as networks (Torok et al., 2018). This is important because network connectivity integrates microstructural effects e.g., neurodegeneration of gray and white matter, or toxicity associated with Aß presence. We hypothesize that network approaches are sensitive to subtle changes arising from the interplay of several factors. While each effect may be small, the summed effect due to individual biomarkers may be significant. Here, we will generate a framework for integrating biomarkers using multimodal approaches (Wiesmann et al., 2016), thereby allowing us to better predict their pathological significance.

To help understand the mechanisms through which APOE genes and their products differentially modulate the brain milieu and circuits to switch from healthy to pathological aging, we use novel mouse models for the APOE4 associated genetic risk. We analyze behavioral and imaging markers including structural connectomics based on high resolution diffusion weighted imaging (DWI) to help understand the underpinnings of network vulnerability in aging and AD (Fischer et al., 2015).

The animal models are homozygous targeted replacement mice, expressing instead of the mouse protein the human APOE3 and APOE4 isoforms. To model the human immune response to age and disease associated challenges these double-transgenic mice only express human NOS2 gene products. This modification enables nitric oxide (NO) production and immune activity regulated by NO to better mimic the human response. Our study includes 12 months old male and female APOE3HN (APOE3/3 + human NOS2 on a mouse Nos2^–/–^ background), and APOE4HN (APOE3/3 + human NOS2 on a mouse Nos2^–/–^ background). Mice were characterized with a behavioral battery for memory function, and with MRI to determine selective vulnerability using regional atrophy and DTI parameters. To these tests we added connectopathy biomarkers extracted using novel statistical approaches that map brain circuits associated with selective vulnerability or resilience conferred by APOE genotypes. While limited in sample size, our study revealed sex specific differences were also present in the networks associated with genotype differences. Our efforts will help identify potential targets for interventions, and future efforts to build models that explain the influence of APOE genotypes on age, sex, and AD associated circuit vulnerability.

## Materials and Methods

### Animals

Using mouse models, we sought to identify vulnerable brain circuits associated with memory dysfunction typical of pathological aging, and with the highest known genetic risk for LOAD - the presence of APOE4 genotype relative to APOE3 genotype. To better model the APOE4 associated risk in humans with AD we have used mouse models named huAPO3/HN and huAPOE4/HN. In these mice, the human NOS2 gene replaced the mouse Nos2 gene (HuNOS2^+/+^/mNos2^–/–^; abbreviated HN). More similar to humans, HuNOS2^+/+^/mNos2^–/–^ mice show unique redox characteristics compared to mice expressing either mNos2, or mNos2 knockouts. To “add-in” the impact of APOE genotype on generation and expression of AD-like pathology, these novel mouse strains co-express HuAPOE3 or HuAPOE4 but on the HuNOS2 background described above. The total number of mice used was 10 APOE3HN mice (4 females, 6 males), and 14 APOE4HN mice (7 males and 7 females), aged to 12 months.

### Behavior Testing

#### The Morris Water Maze

Mice were handled for 5 days prior to the beginning of behavioral testing for the purpose of habituation to the researchers performing the tests. Morris water maze (MWM) was conducted for 5 days, followed by a novel object recognition (NOR) test (2 days).

The MWM tests a mouse’s spatial memory and learning based on their preference for standing on solid ground, as opposed to swimming. Mice are placed in a quadrant of a pool with directional cues and are expected to find a clear platform underneath the water, on which they may stand. Because of their aversion to swimming and the consistent placement of the platform, mice are expected to learn that the platform is located in the same position relative to directional cues and locate it more and more quickly over time. We assessed learning by measuring the amount of time a mouse swam, the distance it swam in the pool, and the percent of the swim time, and swim distance in the target quadrant in which the platform is located (termed target swim time and target swim distance, respectively). The MWM apparatus was a circular pool with 122 cm diameter, and behavior was tracked using with a ceiling-mounted Logitech camera, and analyzed with the video analysis software ANY-maze (Stoelting, Wood Dale, IL, United States). Black mice were allowed to swim in transparent water and were expected to find a glass platform (similar indices of refraction do not allow for easy visibility) located in the south west (SW) quadrant of the pool. Mice were trained for 5 days undergoing four trials each day. For each trial, mice were placed in one quadrant of the maze and had to swim to a 10 cm wide circular platform submerged 1.5 centimeters below the surface of the water (not visible). Each trial consisted of placing the mouse into the water at one of four different starting positions, one in each quadrant and allowing them to swim freely for 1 min. The time needed for the mice to find the hidden platform was recorded as well as the swim path length. If they were unable to locate the platform within the allotted time, they were guided to the platform and allowed to remain there for 10 s. Probe trials were conducted on days 3 and 5, 1 h after the last training trial. During the probe trial the submerged platform was removed and mice were given 1 min to swim in the pool. The amount of time spent in the previous location of the target zone was recorded.

#### Novel Object Recognition

The NOR test assesses a mouse’s memory through exploration. Mice traditionally spend more time exploring novel stimuli, so when they are faced with a stimulus that is novel and one that is familiar, they are expected to remember the familiar object and spend more time exploring and engaging with the more novel object. The day before testing, mice were placed in a 40 cm square open field arena for 5 min to habituate them to the apparatus and the test room. 24 h after habituation, mice were acclimated in the test room for 1 h before beginning trials. Mice first completed an acquisition trial, in which they were placed in the apparatus with two identical objects for 5 min. After a 90 min retention period, mice were then placed in the arena again for 5 min with two dissimilar objects - one that is familiar, and one that is novel. 24 h later, the mice were placed in the arena again for 5 min with a pair of dissimilar objects - one that is the original familiar object, and one that is novel. After each trial, the mouse was returned to its cage. Between trials, the apparatus was cleaned with ethanol solution to eliminate animal clues. The amount of time spent exploring the novel object and the amount of time spent exploring both objects were measured. From this we calculated a recognition index as the time exploring novel object/(time exploring novel object + time exploring familiar object) × 100%. The location preference was similarly calculated, but for two identical objects.

Statistical analyses for behavior tasks was done in JMP (SAS, Cary, NC, United States)^1^. Analysis for multiple measurements acquired in the same animal over time was performed by repeated measures two-way ANOVA using linear mixed models fixed effects for genotype and time and random effects for animals. Tukey HSD was used for *post hoc* corrections. 2-group comparisons used a two-tailed *t*-test, while comparisons between three or more trials were done using a one-way ANOVA. *P* < 0.05 was considered significant.

### Imaging

Brain specimens were imaged on a 9.4 T, 8.9 cm vertical bore Oxford magnet, with shielded coils, providing gradients up to 2000 mT/m (Resonance Research, Inc., Billerica, MA, United States), and controlled by an Agilent Direct Drive Console (Agilent Technologies, Santa Clara, CA, United States). In house made solenoid coils (13 mm diameter) were used to image brain specimens within the skull, in order to avoid tissue damage and distortions. To prepare actively stained brain specimens the animals were anesthetized to a surgical plane and perfused through the left cardiac ventricle, with outflow from the right atrium. Saline (0.9%) was used to flush out the blood, at a rate of 8 ml/min, for ∼5 min. For fixation we used a 10% solution of neutral buffered formalin phosphate containing 10% (50 mM) Gadoteridol (ProHance, Bracco Diagnostics Inc., Monroe Township, NJ, United States), at a rate of 8 ml/min for ∼5 min. Gadoteridol reduced the spin lattice relaxation time (T1) of tissue to ∼100 ms. Mouse heads were stored in 10% formalin for 12 h, then transferred to a 0.01 M solution of phosphate buffered saline (PBS) containing 0.5% (2.5 mM) Gadoteridol, at 4°C for ∼30 days to rehydrate the tissue. Extraneous tissue around the cranium was removed prior to imaging, and specimens were placed in MRI-compatible tubes, immersed in perfluoropolyether (Galden Pro, Solvay, NJ, United States) for susceptibility matching.

We used a diffusion weighted MR imaging to derive microstructural and connectivity information. Our protocol used compressed sensing DWI with an acceleration factor of 4, allowing for efficient sampling and reconstruction in a high performance computing cluster environment (Anderson et al., 2018b; Wang et al., 2018). The DWI protocol used 46 diffusion weighted acquisitions, interwoven with 5 non-diffusion-weighted scans, and the following parameters: TE 12 ms, TR 90 ms, BW 125 kHz, *b* ≈ 4000 s/mm^2^, diffusion pulse width 4 ms, separation 6 ms, amplitude 130.67 G/cm. Images were acquired over a 22 × 11 × 11 mm field of view, with a matrix 368 × 184 × 184, over 14 h, and reconstructed at 55 μm isotropic resolution.

### Image and Network Analysis

Images were processed using a high-performance computing pipeline (Anderson et al., 2017, 2018a,b), to perform diffeomorphic mapping of a symmetric mouse brain atlas, containing 332 regions, based originally of the one presented in Calabrese et al. (2015). To perform these processes we employed at the core of our pipeline advanced normalization tools (Avants et al., 2008, 2011). Each brain was thus segmented in 332 regions. Regional and voxel wise analyses were conducted as in Badea et al. (2019). The Statistical Parametric Mapping SPM toolbox, version 12 (Friston et al., 1994) was used with cluster false discovery rate correction.

We have implemented code for tract based analyses^2^. The tracts connecting pairs of atlas regions (Anderson et al., 2018a) were used to build connectomes based on a constant solid angle (Q-Ball method) method implemented in DIPY (Garyfallidis et al., 2014). We used a relative peak ratio of 0.5, separation angle 25°, and 4 parallel compute threads. We used local tracking with 1 seed per voxel in the whole brain mask, and 0.5 step size. We saved 10% of the 3,000,000 tracks, in trk files of ∼1.5 GB, and their computations required about 20 min/brain using an iMac Pro with 3 GHz Intel Xeon W, 10 cores, with 128 GB memory. Tracts were visualized using DIPY.

Tracts from individual brains were clustered based on a Euclidian distance metric minimization (Garyfallidis et al., 2012), then registered (Garyfallidis et al., 2015, 2018) to a reference brain, before being once more clustered in the space for each specific population (APOE3HN, and APOE4HN).

We hypothesized that genotype and sex modulates network properties, and that we can identify vulnerable circuits relevant to AD. Subnetwork changes were derived using a recently proposed method (Zhang et al., 2019), called tensor network PCA or TNPCA, which is a semi-symmetric tensor generalization of PCA. In short, this works with a tensor network *X*∈*ℝ*^*PxPxN*^, given by the concatenation of the adjacency matrices *Ai*∈*ℝ*^*PxP*^for *i* = 1, …N, where P is the number of nodes (atlas regions), and *N* is the number of subjects. Zhang et al. (2019) estimated a CP model for the semi-symmetric tensors (*X*∈*ℝ*^*I*1*xI*2…*I*..*N*^) by solving:

$$\min_{dk,vk,uk,}{||X-\sum_{k=1}^{K}d_{k}\,v_{k}\circ v_{k}\circ u_{k}||}_{2}^{2}$$

subject to

$$u_{k}^{T}\,u_{k}=1,v_{k}^{T}\,v_{k}=1,v_{k}^{T}\,v_{j}=0,j<k$$

where *v*_*k*_ are P sized vectors, constrained to have orthogonal columns, *u*_*k*_ are N sized vectors, and *d*_*k*_ are CP scaling parameters. In our context *u*_*k*_ denotes the subject mode, and $v_{k}^{T}\,v_{k}$ the network mode.

**FIGURE 1:**
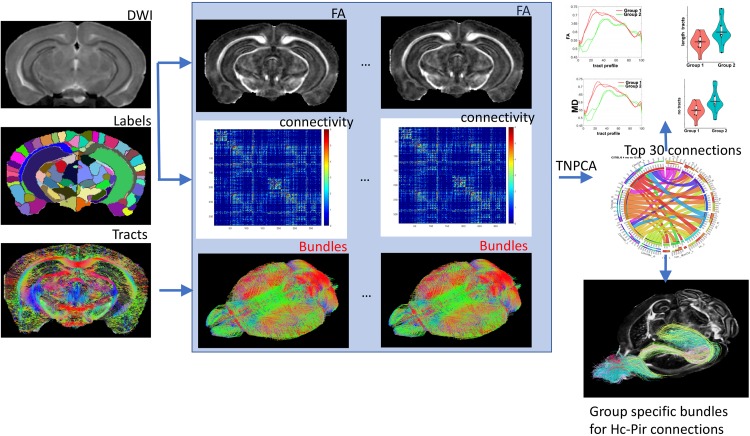
The main elements of our flowchart for characterizing differences between mouse models based on connectivity included image reconstructions and coregistration of individual DWI acquisitions, brain parcelation in 332 regions, and connectome reconstruction based on a constant solid angle method. TNPCA was used to derive subgraphs discriminating two genotypes and the resulting selected pairwise connections between nodes were analyzed for tract length and FA differences. The hippocampus-piriform connections are shown as an example. Hc: hippocampus; Pir: piriform cortex.

The subject modes provides a low dimensional embedding of the connectome for each subject, and can be associated with traits (genotype/phenotypes). The weighted sum of network modes *d*_*k*_ ° *vk* ° *uk*provides a principal brain network which captures the most variation across the population. Thus {*vk* ° *v*_*k*_} can be seen as basis networks, *u*_*k(i) *_ are the normalized coefficients for each subject *i*, and *d*_*k*_ are the scaling factors. We are interested in how the connectome varies across levels of the trait, and for discrete cases such as the genotype, the problem can be approached using linear discriminant analysis, while for continuous cases the problem can be approached using canonical correlation. We used 15 principal components identified from TNPCA, and the projection weights from a Fisher linear discriminant to estimate the top 30 pairwise connections, discriminating amongst our groups/genotypes. These were further analyzed for differences in bundles length and fractional anisotropy (FA). The overall process is detailed in Figure 1.

We used Quick bundles (Garyfallidis et al., 2012) for a more spatially refined analysis based spatial clustering with a distance of 2 mm, and focused on the top 6 subbundles, for comparing along the tract properties between genotypes and sexes. Bundle statistics were evaluated using R^3^.

## Results

We have phenotyped a novel mouse model of genetic risk for LOAD using behavior, regional and voxel based MRI analyses, and network connectopathies based on a recently published dimensionality reduction method called tensor network factorization. Regional and voxel based analyses pointed to overlapping sets of regions affected by atrophy and with lower FA indicative of different microstructural properties. Our results indicated that even though qualitative differences between representative animals of the two groups were subtle, we could separate population groups by genotype based on the lower dimensional representation relying on the tensor network decomposition. Our results identified subgraphs of connected vulnerable regions, and these included areas known to be involved in memory function (e.g., hippocampus), as well as in sensory motor functions (e.g., olfactory areas, and cerebellum).

### Learning and Memory Deficits

**FIGURE 2:**
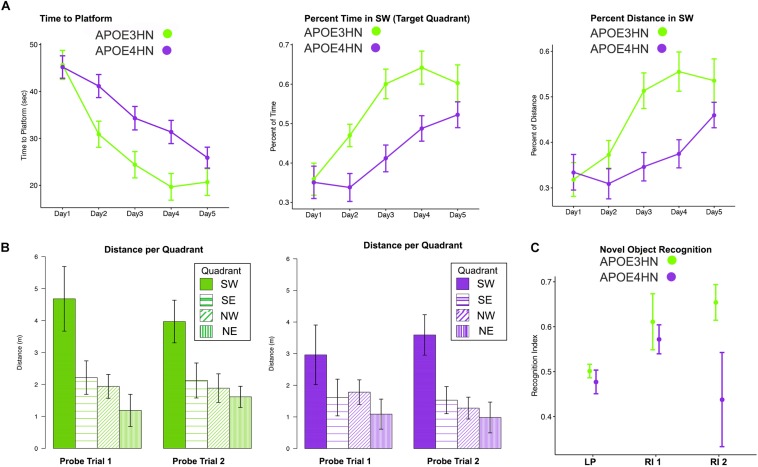
Main repeated measured ANOVA (RMANOVA) results for the memory testing based on acquisition performance and probe trial results (mean ± SEM) in the Morris Water Maze indicate that both APOE3HN and APOE4HN mice learn but there is a significant effect of genotype for both swim time and swim distance. As swim time and distance to hidden platform decreased, the percentage of time spent and distance swam in the target quadrant increased **(A)**. The probe trials indicated that both genotypes had a preference for the SW target quadrant, but APOE3HN mice spent more time swimming in the SW quadrant than APOE4HN mice in the first probe trial **(B)**. E = northeast, NW = northwest, SE = southeast, SW = southwest (target quadrant). *N* = 11 APOE3HN, *N* = 14 APOE4HN mice. **(C)** A novel object recognition test revealed that animals had equal location preferences (LP), and object recognition indices (RI) 90 min later, however, after 24 h APOE4HN mice had lower recognition indices relative to APOE3HN mice (*t* = –2.28, *p* = 0.04). *N* = 10 APOE3HN, *N* = 6 APOE4HN mice (some APOE4HN were not be tested to preserve the matched ages for MRI). Data show mean values, and standard error bars.

Since memory is expected to be deficient in animal models of AD, we tested both spatial and NOR memory in 14 APOE4HN and 11 APOE3HN animals (one died before being imaged). Spatial memory was examined through acquisition and probe trials in the MWM (Figures 2A,B). Swim time (and distance) to the hidden platform got shorter with time for both groups. Repeated measures ANOVA (RMANOVA) detected a significant effect of day *F*(4,92) = 26.1, *p* < 0.001 (Figure 2A), and genotype [*F*(1,23) = 6.3, *p* < 0.02], while the interaction term of day by genotype was *F*(4,92) = 2.0, *p* < 0.09. For swim distance there was a significant main effect of day *F*(4,83.8) = 34.4, *p* < 0.0001, and a significant day × genotype interaction with *F*(4,83.8) = 3.6, *p* < 0.01. Within genotypes there was a significant difference after Tukey HSD tests for swim distance for APOE3HN mice between days 1 and 2 (*t* = 3.5, *p* < 0.02); 1 and 3 (*t* = 6.3, *p* < 0.0001); 1 and 4 (*t* = 7.3, *p* < 0.0001); 1 and 5 (*t* = 8.5, *p* < 0.0001); 2 and 4 (*t* = 4, *p* < 0.006); 2 and 5 (5.2, *p* < 0.0001). For genotype APOE4HN these differences were significant between days 1 and 4 (*t* = 3.8, *p* < 0.01); 1 and 5 (*t* = 5.8, *p* < 0.0001); 2 and 4 (*t* = 3.8, *p* < 0.01); 2 and 5 (*t* = 5.7, *p* < 0.0001); 3 and 5 (*t* = 3.3, *p* < 0.04). No differences were noted between days 4 and 5.

We have measured the percent time spent in the target quadrant during learning trials and found a significant effect of day [*F*(4,92) = 14.3, *p* < 0.0001] and genotype [*F*(1,23) = 15.8, *p* < 0.0006], with a possible interaction term (*p* < 0.1). At day 3 the difference between genotypes was largest (*t* = 3.5, *p* = 0.02). For the percent distance swam in the target quadrant during the learning trials we found a significant main effect for day [*F*(4,83.7) = 13.8, *p* < 0.0001], for genotype [*F*(1,21.7) = 15.7, *p* < 0.0007] and a significant interaction [*F*(4,83.7) = 3.6, *p* < 0.01]. The differences with genotype were significant for days 3 (*t* = 3.6, *p* < 0.02); and persisted for day 4 (*t* = 3.9, *p* < 0.008). Differences subsided by day 5.

The first probe trial was performed on the third day and indicated a significant effect of quadrant only (*p* < 0.0001). APOE3HN mice had a significant preference for the SW quadrant relative to the SE (*t* = 3.9, *p* = 0.006), NE (*t* = 5.3, *p* < 0.0001), NW (*t* = 4.1, *p* = 0.003), but not for SE. APOE4HN mice preferred the target SW quadrant over SE (*t* = 4, *p* = 0.004) and NE quadrants (*t* = 4.3, *p* = 0.001), but only reached a trend for NW (2.8, *p* = 0.1).

The first probe distance swam in the target quadrant provided a more sensitive marker for the memory deficits, showing significant genotype (*p* < 0.003), and quadrant effects (*p* < 0.0001). Genotypes had significant differences, with APOE3HN mice swimming longer in the SW than APOE4HN mice (*t* = 3.74, *p* = 0.008). APOE3HN preferred the SW relative to NE (*t* = 6.9, *p* < 0.0001), NW (*t* = 5.4, *p* < 0.0001), SE (*t* = 4.9, *p* = 0.0002). APOE4HN also preferred the SW over NE (*t* = 4.2, *p* = 0.002), and differences reached a trend relative to SE (*t* = 3, *p* = 0.07), but they made no distinction relative to NW.

The second probe swim times performed on the fifth day also showed an effect of the quadrant (*p* < 0.0001), but not for genotype. APOE3HN mice preferred the SW to NE (*t* = 3.8, *p* < 0.006), NW (*t* = 4.3, *p* = 0.03), SE (4.3, *p* = 0.06). APOE4HN mice also preferred the SW to the NE (*t* = 3.8, *p* < 0.0001), NW (*t* = 4.8, *p* < 0.002), SE (*p* = 3.1, *p* = 0.05).

The second probe swim distance showed a significant effect of quadrant (*p* < 0.004), and genotype (*p* < 0.0001). Between genotypes the swim distance in SW was not significantly different. APOE3HN mice swan longer distance in the SW relative to NE (*t* = 5.9, *p* < 0.0001), NW (*t* = 5.2, *p* = 0.0001), SE (4.6, *p* = 0.0004). APOE4HN mice also preferred the SW to the NE (*t* = 6.3, *p* < 0.0001), NW (*t* = 5.6, *p* < 0.0001), SE (*p* = 5, *p* = 0.0001) (Figure 2C).

During the NOR APOE4HN and APOE3HN mice showed no location preference for the sites of the two objects presented. The immediate recognition index was not different between genotypes. After 90 min, however, APOE4HN mice showed more similar preferences for the familiar and novel objects (RI = 0.44 ± 0.07 (SE), CI = [0.28. 0.60]) relative to APOE3HN mice (0.65 ± 0.06 (SE), CI = [0,53 0.78]). This indicated that APOE4HN mice did not remember the familiar object used during the acquisition trial. APOE3HN mice had a higher recognition index compared to APOE4HN mice at 24 h after the initial trial (*t* = 2.3, *p* = 0.04)].

### Volume Loss

**FIGURE 3:**
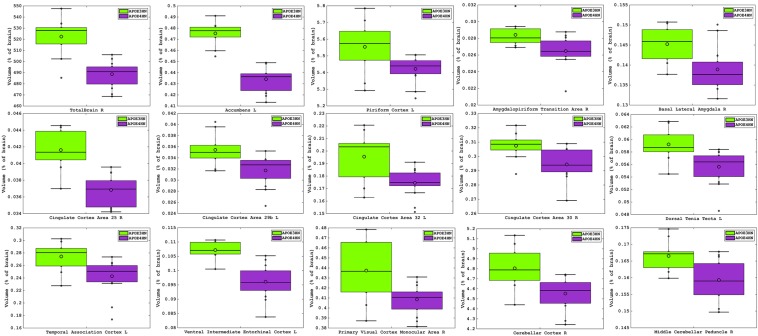
Volume atrophy was detected in regions spanning from the rostral to the caudal aspects of the brain, and ranged from 10% for the temporal association cortex, entorhinal and cingulate cortex, down to 2% for the piriform cortex. The visual cortex, accumbens and amygdalo-piriform transition areas were ∼7% smaller and the cerebellum was ∼5% smaller in APOE4HN mice, FDR = 5%.

The total brain volume for APOE4HN mice was 6% smaller relative to APOE3HN controls. An ROI (region of interest) analysis for the 332 brain parcelation revealed significant atrophy occurred for regions shown in Table 1 and Figure 3.

**TABLE 1:** Volume atrophy was observed at the level of the whole brain (mm^3^) in APOE4HN mice relative to APOE3HN mice, and in select regions (volumes are reported for one hemisphere, as % of total brain volume).

**Structure**	**APOE4HN**	**APOE3HN**	**pFDR**	**CI[1]**	**CI[2]**	***t***	**Cohen *d***	**Diff (%)**
	**(mean ± SD)**	**(mean ± SD)**						
Temporal_association _cortex (%)	0.243 ± 0.028	0.274 ± 0.023	2.67E-02	−0.054	−0.009	−2.93	−1.21	−11.53
Cingulate_cortex _area_25 (%)	0.037 ± 0.002	0.042 ± 0.002	2.68E-04	−0.007	−0.003	−5.64	−2.34	−11.50
Cingulate_cortex _area_32 (%)	0.175 ± 0.011	0.195 ± 0.02	1.31E-02	−0.034	−0.008	−3.32	−1.37	−10.68
Cingulate_cortex _area_29b	0.032 ± 0.003	0.035 ± 0.003	1.48E-02	−0.006	−0.001	−3.26	−1.35	−10.46
Ventral_intermediate _entorhinal_cortex	0.096 ± 0.006	0.107 ± 0.003	3.20E-04	−0.015	−0.007	−5.51	−2.28	−10.30
Accumbens	0.434 ± 0.011	0.475 ± 0.011	2.43E-06	−0.051	−0.032	−9.04	−3.74	−8.67
Cingulate_cortex_area _24b_prime	0.054 ± 0.003	0.058 ± 0.004	1.61E-02	−0.008	−0.002	−3.22	−1.33	−7.91
Secondary_visual_cortex _mediomedial_area	0.192 ± 0.009	0.206 ± 0.016	3.26E-02	−0.024	−0.004	−2.82	−1.17	−6.85
Amygdalopiriform _transition_area	0.026 ± 0.002	0.028 ± 0.001	3.05E-02	−0.003	−0.001	−2.86	−1.18	−6.83
Primary_visual_cortex _monocular_area	0.409 ± 0.015	0.437 ± 0.032	2.38E-02	−0.049	−0.009	−3.01	−1.25	−6.60
Cingulate_cortex _area_29c	0.181 ± 0.008	0.193 ± 0.008	7.49E-03	−0.019	−0.005	−3.64	−1.51	−6.35
Dorsal_tenia_tecta	0.056 ± 0.003	0.059 ± 0.003	1.38E-02	−0.006	−0.001	−3.29	−1.36	−6.03
Cerebellar_cortex	4.553 ± 0.157	4.805 ± 0.205	1.10E-02	−0.405	−0.100	−3.43	−1.42	−5.25
Pontine_nucleus	0.126 ± 0.004	0.132 ± 0.006	4.00E-02	−0.010	−0.001	−2.71	−1.12	−4.35
Basal lateral amygdala	0.139 ± 0.005	0.145 ± 0.004	2.29E-02	−0.011	−0.002	−3.04	−1.26	−4.35
Middle_cerebellar _peduncle	0.159 ± 0.006	0.167 ± 0.005	1.79E-02	−0.012	−0.002	−3.17	−1.31	−4.31
Cingulate_cortex_area_30	0.294 ± 0.011	0.307 ± 0.009	1.90E-02	−0.022	−0.004	−3.14	−1.30	−4.24
Piriform_cortex	5.422 ± 0.077	5.554 ± 0.155	3.62E-02	−0.231	−0.033	−2.76	−1.14	−2.37
TotalBrain (mm^3^)	488.64 ± 11.21	522.44 ± 17.52	2.30E-04	−45.94	−21.66	−5.77	−2.39	−6.47

The largest volume loss in APOE4HN mice relative to the APOE3HN controls was in the range of ∼10% and occurred for the cingulate cortex (areas 25, 29b, and 32), the ventral intermediate entorhinal cortex and the temporal association cortex. The accumbens, amygdalo-piriform transition area, and secondary visual cortex were 7% or smaller in APOE4HN mice relative to APOE3HN mice. Finally, the cerebellar cortex, middle cerebellar peduncle and pontine nuclei were ∼4% smaller, while the piriform cortex was 2% smaller.

### Microstructural Integrity

Regional analyses for FA did not survive multiple comparison correction, but there was a trend for the medial lemniscus to have higher FA in APOE4HN carriers (*p* corrected = 0.1). The cerebral peduncle had a 6% lower FA in APOE4HN mice (*p* corrected = 0.1). Similarly, the axial diffusivity differences did not survive the multiple correction, and the longitudinal fasciculus of pons in APOE4HN mice had a 4% lower axial diffusivity (*p* uncorrected = 0.02), and the cerebellar white matter had 6% larger radial diffusivity (*p* uncorrected = 0.02).

### Voxel Based Analyses

**FIGURE 4:**
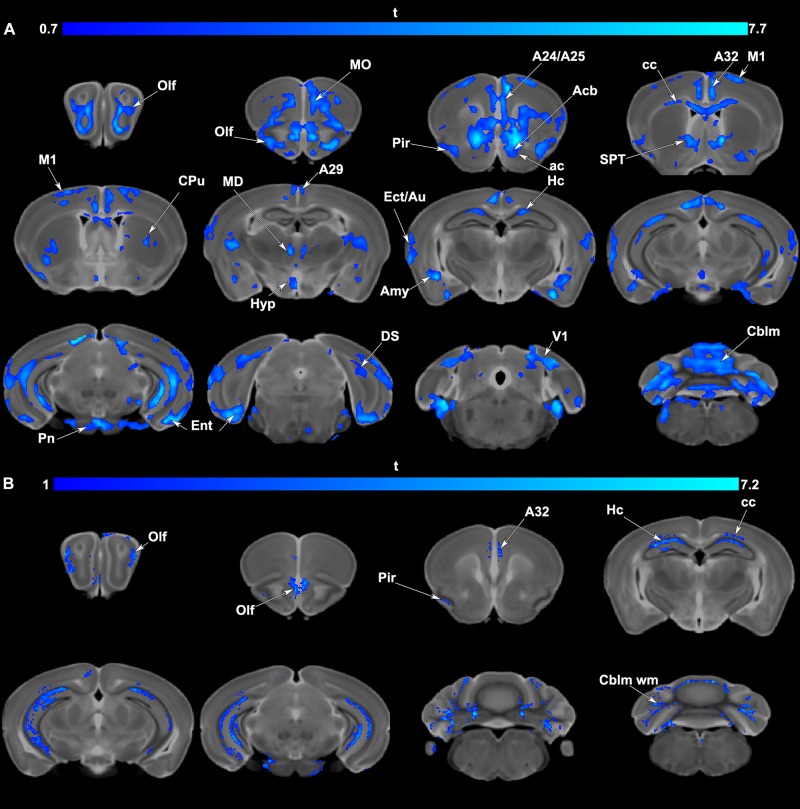
**(A)** Voxel based analyses indicated that volume atrophy occurred in vulnerable regions comprising olfactory/piriform (Olf, Pir) cingulate (A24,25,29, 32), sensory (Ect: ectorhinal, Au: auditory, V1: primary visual cortex) and motor cortex (M1), and the entorhinal cortex (Ent). Deeper gray matter regions with atrophy in APOE4 carriers included the accumbens (Acb), caudate putamen (CPu), hippocampal formation (Hc, subiculum: DS), amygdala (Amy), as well thalamic nuclei (mediodorsal: MD) and the cerebellum (Cblm) and pontine nuclei (Pn). Among white matter tracts the anterior commissure (ac), and corpus callosum (cc) also had areas of atrophy. Results are presented as t maps, FDR cluster-corrected for multiple comparisons, using an initial cluster forming threshold of 0.05 significance, and the whole brain as a mask (blue color). **(B)** Voxel based analyses indicative of fractional anisotropy (FA) reductions suggested vulnerable brain networks. These included the olfactory (Olf) and in particular the piriform cortex (Pir), cingulate cortex (A32), hippocampus (Hc), and the white matter of the corpus callosum (cc) and cerebellum (Cblm wm). Results are presented as t maps, FDR cluster-corrected for multiple comparisons, using initial cluster forming threshold of 0.05 significance, and the whole brain as a mask (blue color). The DWI minimum deformation average template serves as the background.

Voxel based analyses indicated significant volume (Figure 4A) and FA (Figure 4B) reductions occurred in APOE4 carriers relative to APOE3 carriers. Areas of atrophy included the olfactory cortices, hippocampus, subiculum, cingulate cortex, amygdala and entorhinal cortex, as well as the cerebellum. Sensory motor cortex areas also suffered atrophy. Areas with FA reductions were less extensive than those with volume atrophy and were noted in the olfactory/piriform and cingulate cortices, hippocampus and cerebellum.

### Connectopathies

The tensor network analysis revealed the top connected subnetwork (Table 2) differentiating the two genotypes. The top 30 resulting connected subnetworks included predominantly interhemispheric connections, and 7 distinct anatomical regions: the hippocampus, piriform cortex, cerebellum (white matter and gray matter), the caudate putamen/striatum, gigantoreticular nucleus and the corpus callosum. Intrahemispheric connections pointed to a role for the cerebellum. The three most frequent major structures included the piriform cortex, the hippocampus, and the cerebellum.

**TABLE 2:** The top connections for the subnetworks differentiating between APOE3HN and APOE4HN connectomes.

	**Connection**	**Load**		**Connection**	**Load**
1	Hippocampus_right—cerebellar_cortex_left	92.49	16	Cerebellar_cortex_right—corpus_callosum_left	73.48
2	Cerebellar_white_matter_left—cerebellar_cortex_left	–91.78	17	Cerebellar_cortex_right—cerebellar_white_matter_left	–73.3
3	Piriform_cortex_right—cerebellar_cortex_left	91.36	18	Cerebellar_cortex_left—piriform_cortex_left	72.87
4	Cerebellar_cortex_right—hippocampus_right	89.09	19	Cerebellar_white_matter_left—hippocampus_left	69.75
5	Cerebellar_cortex_right—piriform_cortex_left	89.04	20	Corpus_callosum_right—cerebellar_cortex_right	67.61
6	Cerebellar_white_matter_right—cerebellar_cortex_right	–84.69	21	Corpus_callosum_right—cerebellar_white_matter_left	67.04
7	Cerebellar_white_matter_right—hippocampus_left	82.28	22	Gigantocellular_reticular_nucleus_right—piriform_cortex_left	–64.24
8	Cerebellar_cortex_right—piriform_cortex_right	80.79	23	Gigantocellular_reticular_nucleus_left—piriform_cortex_left	–63.63
9	Hippocampus_right—piriform_cortex_left	–80.58	24	Cerebellar_cortex_left—hippocampus_left	61.36
10	Cerebellar_white_matter_right—piriform_cortex_left	79.61	25	Cerebellar_white_matter_right—corpus_callosum_left	61.18
11	Piriform_cortex_right—cerebellar_white_matter_left	78.76	26	Cerebellar_cortex_right—striatum_left	61.04
12	Piriform_cortex_right—hippocampus_left	–77.31	27	Cerebellar_white_matter_left—piriform_cortex_left	60.3
13	Cerebellar_cortex_right—hippocampus_left	76.73	28	Cerebellar_white_matter_right—piriform_cortex_right	60.19
14	Corpus_callosum_right—cerebellar_cortex_left	76.46	29	Striatum_right—cerebellar_cortex_left	59.53
15	Cerebellar_white_matter_right—cerebellar_cortex_left	–74.67	30	Corpus_callosum_left—cerebellar_cortex_left	58.95

**FIGURE 5:**
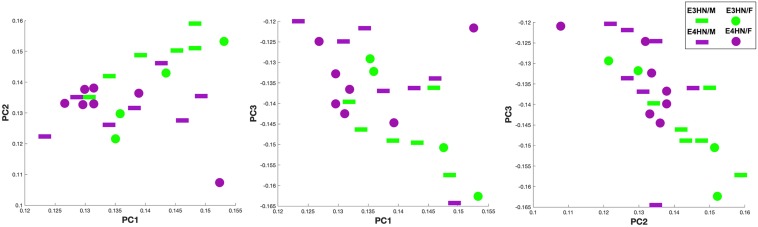
Scatter plot of the top three principal components for the connectome TNPCA analysis. The two genotypes are shown in green: APOE3HN, and purple: APOE4HN. Sex information is also indicated, although sex was not used as a predictor (female: disk, male: bar).

Figure 5 shows the scatter plot for the top three principal components, which explained 61% percent of the variation, while the top 15 explained 91% of the variation between genotypes. We selected examined the same graphs to identify whether sex differences were also apparent within genotypes, but these differences were less clear in our small sample.

**FIGURE 6:**
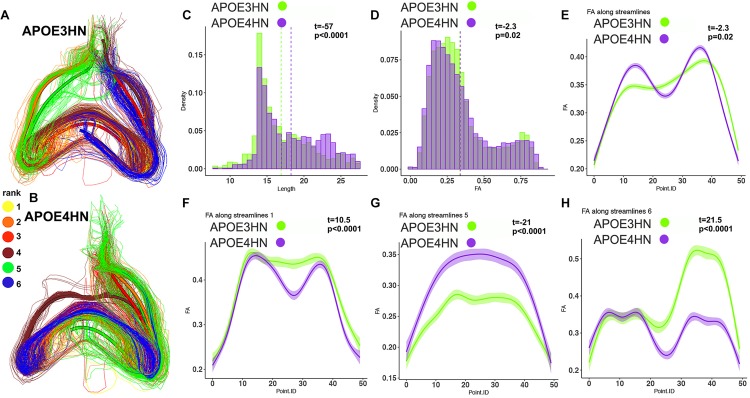
The piriform cortex-hippocampal interhemispheric connections through the top 6 bundles were ranked according to size (yellow for the first and largest bundle, orange for the second, red for the third, brown for the fourth, green for the fifth, blue for the sixth. The interhemispheric connections appeared stronger in the APOE3HN mice relative to APOE4HN mice **(A)** and **(B)**, according to the size based ranking for the major sub-bundles. This indicated different connectivity patterns for the two genotypes. Differences in fiber length distributions between the two genotypes are shown in **(C)**, and in FA distributions in **(D)** using histogram densities. These indicate a slight shift toward longer length **(C)**, but lower FA values in APOE4HN mice, which may suggest dismyelination **(D)**. After establishing spatial correspondence through an affine bundle centroid registration, we detected that differences along the bundle containing all connections between the piriform cortex and hippocampus were not uniform **(E)**. We identified the top 3 sub-bundles accounting for the largest difference between the genotypes **(F–H)**. FA appeared in general lower for APE4HN mice in sections of two of these subbundles **(F,H)**, but higher in one subbundle **(G)**.

We selected examples among the top ranked connections, featuring the most frequent regions. We observed that the interhemispheric connectivity between the hippocampus and piriform cortex (ranked 9) was stronger in APOE3HN mice relative to APOE4HN mice, as illustrated qualitatively in Figures 6A,B showing the top 6 largest bundles, ranked according to their size. Figures 6C–E compare the distributions of fiber length, FA, and FA along the whole bundle set for the two genotypes. Figures 6F–H compare the FA distribution along spatially matched subbundles between genotypes, indicating that FA is non-uniform along the bundles.

**FIGURE 7:**
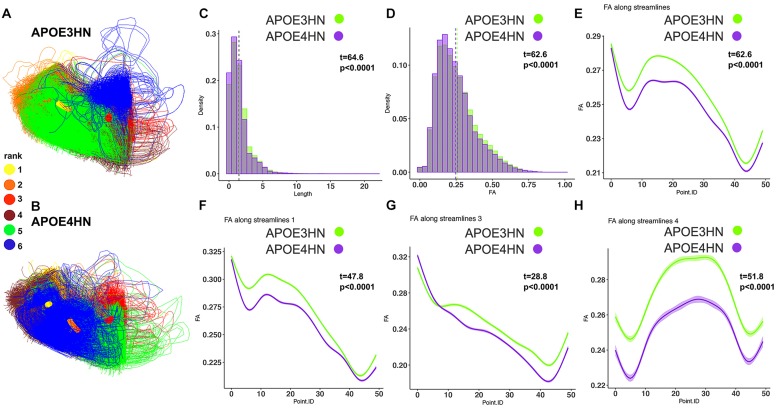
The 2nd ranked connection discriminating between the genotypes involved intrahemispheric cerebellar connections between white and gray matter. APOE4HN **(B)** showed consistent deficiencies relative to APOE3HN **(A)** carriers in fiber length and FA distributions **(C,D)**. These differences were evident in whole bundle **(E)** and subbundle analyses **(F–H)**.

The second ranked connection pertained to the intracerebellar connectivity, and APOE4HN mice had consistently shorter connections, and lower FA along the bundles; both when analyzing the connectivity of the two nodes, as well as along the significant sub bundles (Figure 7).

**FIGURE 8:**
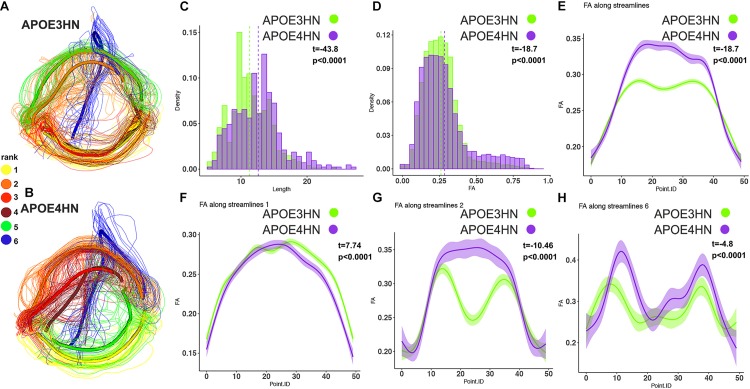
The interhemispheric hippocampal cerebellar connections showed significant differences between APOE3HN **(A)** and APOE4HN **(B)** mice in length **(C)** and FA **(D,E)**. The top three subbundles with significant differences between genotypes showed lower FA for APOE4HN mice relative to APOE3HN in the largest subbundle **(F)**, but higher FA for the 2nd and 6th spatially matched subbundles **(G,H)**, and also higher variability for the APOE4HN genotype.

The third example shows the interhemispheric connection between the hippocampus and cerebellum (Figure 8), which was the top ranked connection discriminating between genotypes. Distinct bundles showed larger FA in APOE4HN (overall, and in subbundles 2 and 6), while the largest subbundle (1) showed higher FA for the first portion of the bundle but lower FA for the second half.

**FIGURE 9:**
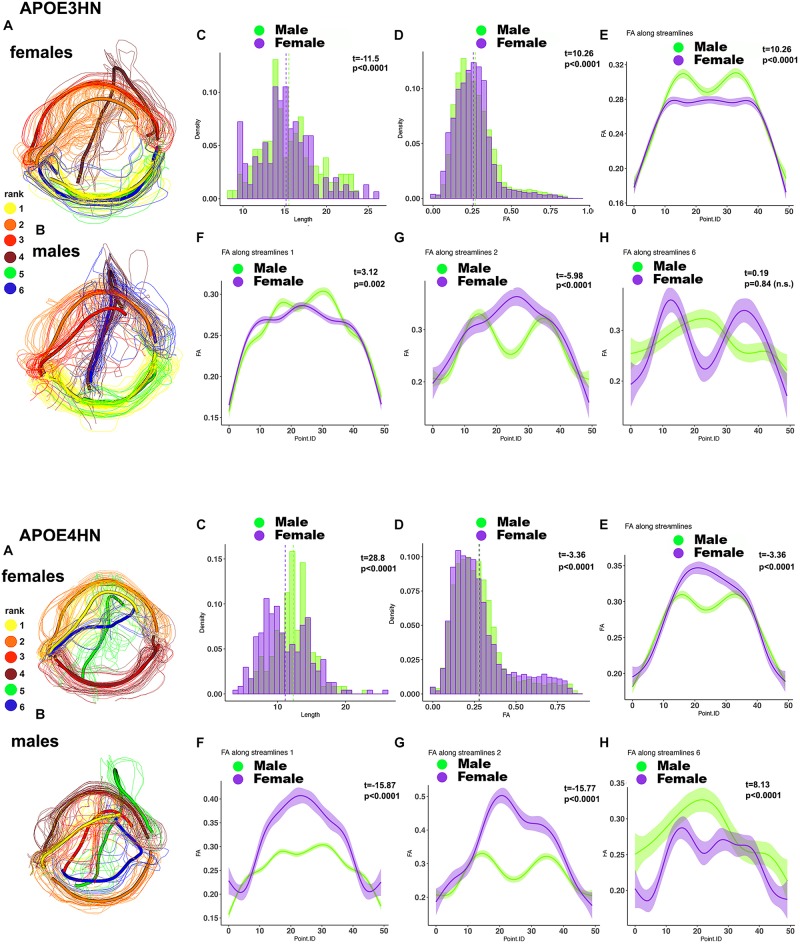
Within genotype, between sex (**A**: female; **B**: male) analyses for the hippocampal-cerebellar interhemispheric connections. APOE3HN females had longer connections compared to males and the opposite was true for APOE4HN mice **(C)**. Differences in FA over the whole set of streamlines **(D)** were subtler in terms of effect sizes, but clearly evident in our along the bundle analysis. APOE3HN males had overall higher FA values than females, and the opposite was true for APOE4HN mice **(E)**. The top subbundles with significant genotype differences **(F)** had also higher FA for APOE3HN males compared to females (bundle 1), while APOE4HN females had higher FA compared to males. The 2nd ranked bundle showed higher FA in females compared to males of both genotypes, with a more accentuated difference for APOE4HN mice **(H)**. The 3rd ranked subbundle did not show sex differences for APOE3HN mice, while APOE4HN males showed higher FA compared to females of the same genotype.

A further analysis of the hippocampal-cerebellar interhemispheric connections revealed within genotype, between sex differences in the length and FA distributions in both APOE3HN, and APOE4HN mice, as well as in the spatially characteristic patterns along the bundles (Figure 9). Sex based differences based on fiber length were smaller in APOE3HN mice, compared to those observed in APOE4HN models. The males appeared to have higher FA along the whole bundle relative to the females in APOE3HN mice, but the opposite was seen in APOE4HN mice. The spatial distribution of these effects was not uniform throughout the brain or along subbundles. This illustrates that sex specific differences may be harder to detect in the absence of detailed bundle analytics performed in spatially aligned bundles.

**FIGURE 10:**
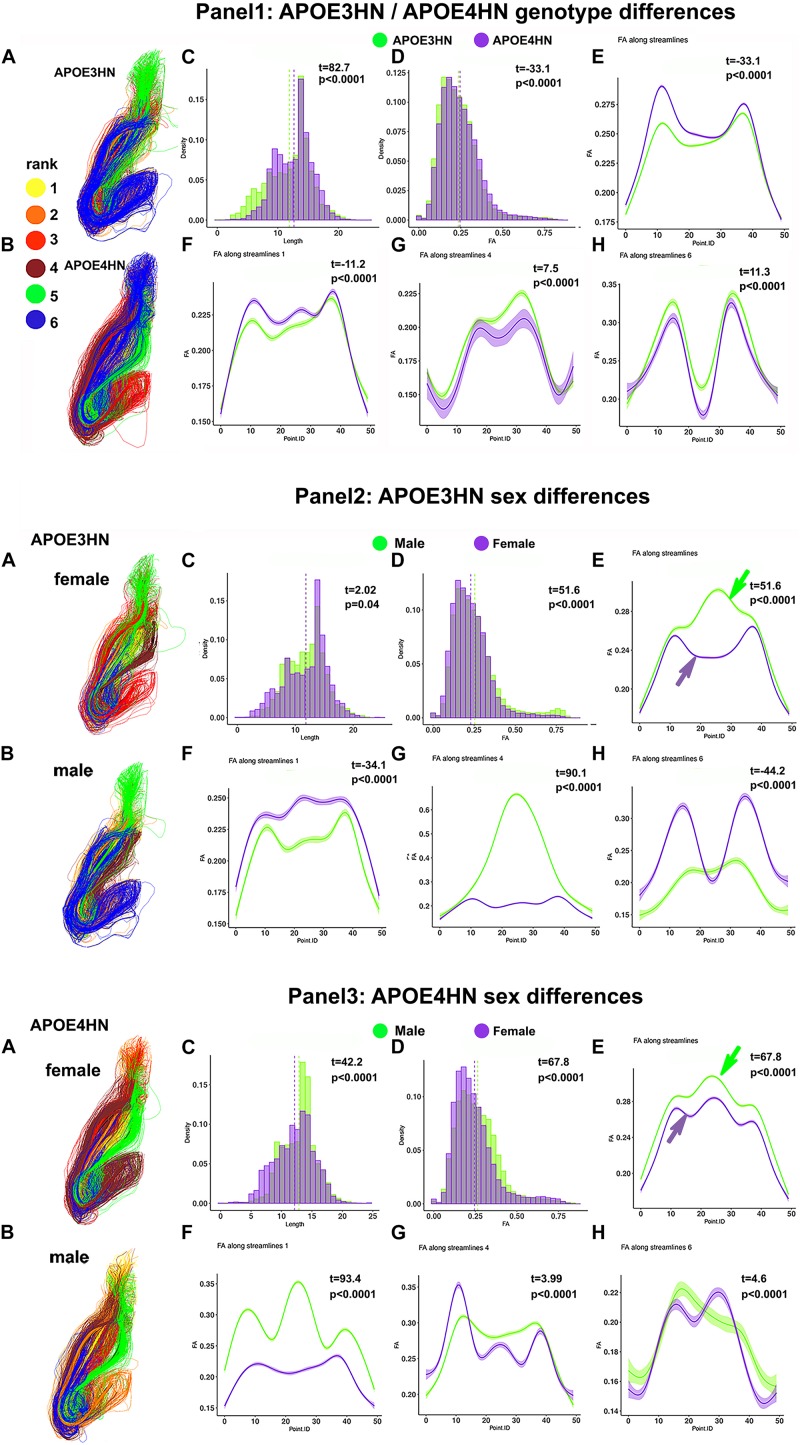
Intrahemispheric connections between the hippocampus and piriform cortex. The first panel compares the two genotypes; the second panel compares the two sexes, within the APOE3HN genotype; the third panel compare the two sexes, within the APOE4HN genotype. Fiber length and FA distributions are shown in **Panels 1–3, C, D**. Qualitatively males of the two genotypes presented more similar, consistent bundle FA shapes, while females showed more variability between the genotypes **(panels 2E,3E)**. Overall, females had lower FA along the entire bundle set in both APOE3HN **(panel 2E)** and APOE4HN mice **(panel 3E)**. Interestingly, APOE3HN females had larger FA than males for the largest subbundle **(panel 2F)**. However, FA was lower along the same subbundle in APOE4HN females relative to males of the same genotype **(panel 3F)**, and differences were larger relative to those between males and females of APOE3HN genotype. These patterns varied by subbundle, and spatially, along the bundles.

We have examined the intrahemispheric connections between the hippocampus and piriform cortex (Figure 10), and observed larger variability within the APOE4HN genotype relative to APOE3HN, as indicated by the width of the confidence intervals (particularly in panel 1G). Qualitatively males of the two genotypes presented more similar/or consistent bundle FA shapes, and females showed more variability in the FA curve shape between the genotypes (panel 2E, and 3E, arrows). Overall, females had lower FA along the entire bundle set in both APOE3HN (panel 2E) and APOE4HN mice (panel 3E). However, we observed lower FA values along the largest subbundle in APOE4HN females relative to males of the same genotype (panel 3F), and larger differences relative to those between males and females of APOE3HN genotype (where females had larger FA overall). We noted a spatially varying pattern of FA changes along bundles, possibly denoting different myelination, or microenvironment properties.

Together, differences in behavioral responses, morphometry, FA and connectivity denote that APOE4HN and APOE3HN mice may use different strategies for learning and memory; and that an association of multiple factors probably contributed to the observed behavioral impairment. We have found that the bundle analysis may confer increased sensitivity to genotype and sex differences, by investigating changes beyond the level of associating the connectivity between two regions with a single entry in the connectome matrix. Our along the bundle analyses revealed rather than a uniform effect, a spatially varying pattern of FA changes along bundles, possibly denoting increased sensitivity to local connectivity, myelination, or microenvironment properties.

## Discussion

There is a rapid growth in the number of people affected by Alzheimer’s disease, yet we do not know its etiology or have effective treatments. To examine factors which contribute to the switch from normal to pathological aging we focused on the APOE polymorphic alleles. The causes for increased risk, or conversely resilience, conferred by the major APOE alleles are not known. The APOE4/4 genotype is the main genetic risk for late onset Alzheimer’s disease (AD), and is associated with a 30–55% risk of developing mild cognitive impairment or AD by age 85, compared to 10–15% for the APOE3/3 genotype.

To help understand the mechanisms through which APOE genes and their products differentially modulate the brain and its circuits, we implemented a multi-disciplinary approach using homozygous targeted replacement APOE3 and APOE4 mice expressing the major human APOE isoforms, under the control of the mouse endogenous ApoE promoter. To model the human immune response to aging we used double-transgenic mice that express human NOS2 gene products. This modification enables NO production and immune activity regulated by NO to better mimic the human response. Mice were characterized with a cognitive behavioral battery for memory alterations typical of AD, and with MRI to determine selective vulnerability of associated brain networks. Our imaging measures were based on volume and DWI; and our analyses of brain connections provided insight into networks properties. We aimed to reveal how APOE genotypes differentially confer vulnerability or resilience to select brain circuits during aging, and for different sexes. Identification of vulnerable networks may help understand the etiology of neurodegenerative disease, and facilitate targeted interventions. Monitoring such changes with sensitive biomarkers may help stratify patients, and assess response to therapies.

Our behavioral tests determined that APOE4HN mice have deficits in the learning and memory function as tested in the MWM during learning trials and during the first probe tests at 3 days, but not at 5 days. The NOR also indicated deficits as the recognition index was lower when tested at 24 h, but not 90 min after the initial objects presentations. These deficits in long term memory for APOE4HN mice suggest perturbations in brain networks involved in memory function.

APOE4HN had 6% smaller brains when compared to age matched APOE3HN controls, and the regions accounting for these differences included the entorhinal and temporal association cortex, the cingulate cortex and amygdala (McGaugh et al., 1996), suggesting alterations in emotional memory in addition to the demonstrated spatial and object recognition memory effects we have measured. Interestingly, the amygdalopiriform transition area, and the accumbens were also smaller in APOE4HN mice. Sensory and motor areas such as the olfactory areas/the piriform cortex, the visual and motor cortex areas, and the cerebellum and its connections also suffered atrophy. These regional changes point to spatially extensive network alterations in APOE4HN mice. Voxel based analyses confirmed these findings and added information due to increased sensitivity to smaller clusters of atrophy in the primary motor cortex, striatum, septum, subiculum, and the mediodorsal thalamic nuclei. We found changes in the volume of the pons and cerebellum, which have been traditionally thought to be involved only in late stages of AD, but have also been shown in age related tauopathy, independently of Aβ presence (Josephs et al., 2017). Fractional anisotropy and connectivity also helped distinguish APOE4 from APOE3 carriers. Interestingly, hyper functional connectivity (Wang et al., 2017) in MCI APOE4 carriers may suggest a compensatory role for the cerebellum at early stages. When present, cerebellar pathology has been associated with increased rates of cognitive dysfunction (Liang and Carlson, 2019), and to be predictive of conversion from MCI to AD. Moreover, in cases of accelerated neurodegeneration, such as chronic traumatic encephalopathy (CTE) following repeated traumatic brain injury (TBI), the cerebellum appears to be one of the most vulnerable brain regions and exhibiting pathology early on (Liang and Carlson, 2019).

This is in contrast with the traditional view associating the cerebellum exclusively with motor coordination and learning, but supported by more recent studies, which have revealed a role for the cerebellum in cognitive functions such as attention, language, working memory, emotion, and in visuospatial navigation (Timmann and Daum, 2007; Baillieux et al., 2008; Timmann et al., 2010). Viral tracer studies have recently demonstrated previously unknown connections between the cerebellum and hippocampus – in particular a polysynaptic circuit from the cerebellar fastigial nucleus with a thalamic relay in the LDDM/LDVL and VL, which in turn synapses on the subiculum, retrosplenial cortex, and rhinal cortex, which all project to the hippocampus (Bohne et al., 2019). These connections are indicative of a role in spatial navigation. Our studies support that APOE4 carriers have differences in such pathways connecting the cerebellum with the hippocampus and also with the piriform cortex, and these differences co-exist with alterations in spatial learning and memory, as well as remote memory for object recognition. Our study suggests that more attention needs to be given to understanding the role of the cerebellum in neurodegenerative diseases, and associated cognitive deficits.

White matter tracts with reduced volume included the corpus callosum, anterior commissure and the middle cerebellar peduncles. FA reductions, commonly seen as indicators of altered microstructural integrity in white matter tracts, were found mostly in the corpus callosum and the cerebellar white matter. In addition hippocampal projection pathways had lower FA, and we noted FA reductions in CA1 areas, where from projections connect to the subiculum and the entorhinal cortex, but also to the basolateral amygdala (BLA), which sends projections to the medial frontal cortex, and the accumbens (also the bed nucleus of stria terminalis, and central amygdaloid nucleus) (Mandyam, 2013).

The complexity of these relationships and the extent of the networks involved demands the development of integrative methods followed by dimensionality reduction strategies. Here we have used a recently developed method (Zhang et al., 2019) for assembling structural connectomes into tensor networks, and mapping those into a reduced dimensional space to identify significant subnetworks associated with traits. This relies on a generalization of principal component analysis. In our case the top 15 principal components explained 91% of the variance. The tensor network principal component analysis helped reveal the top 30 connections, including seven unique structures that best distinguished amongst our two genotypes. A significant portion of these connections were interhemispheric. We found that the pairwise connectivity between two nodes, most often used in standard connectometry studies contain rich information that can be further exploited to reveal genotype and sex differences. The histogram based analyses for tract length and FA were supplemented by bundle specific analyses on spatially clustered sub-bundles, and illustrated different wiring patterns and properties in APOE4HN and APOE3HN, as well as between sexes within each genotype. We paid particular attention to the interhemispheric connections between the hippocampus and piriform cortex, the cerebellum and hippocampus, and the intrahemispheric cerebellar connections. Prompted by the frequency of appearance for the piriform cortex and hippocampus in the top list of connections we also examined the properties of their intrahemispheric connections (Figure 10), and these confirmed the male associated differences between genotypes, while showing a stronger tendency for lower FA along these projections for APOE4HN mice.

The main limitations of this study come from the small sample size, and the fact that we pooled our bundles for statistical analysis rather than stratifying them by animal. We argue this provides a first step approach to study differences with genotypes in animal cohorts which provide virtually genetically identical replicates. Also, formalin fixation may affect tissue properties and cause shrinkage, and we have tried to control these factors by preserving the same interval between animal sacrifice and imaging. Further studies should include more replicates of each sex to infer sex specific interactions between vulnerable networks and APOE alleles.

We note that APOE-HN mice do not express mutated APP leading to prevalent amyloid pathology, therefore our study could not address the mechanism of interaction between the various APOE alleles and Aß. However, the literature suggests that APOE4 exerts an effect on the pathogenesis on AD through Aß and also Aß independent pathways (Huang, 2010). While the deposition of Aβ is apoE isoform-specific, it is not clear whether and how they influence the accumulation and progression of tau pathology (Balu et al., 2019). APOE isoforms also affect neuroinflammation, vascular function, metabolism, synaptic plasticity, and transcription regulation (Liao et al., 2017). In addition to the human APOE alleles, our mouse models have a murine NO synthase 2 knockout background (*mNos2^–/–^*) (Colton et al., 2006, 2014). In place of the mouse Nos2 gene these express a functional human NOS2 gene (Vitek et al., 2006). These modifications lead to reduced immune-activated *NOS2* expression and *iNOS* production compared to wild type rodents. This allows to model the human innate immune response, in particular with respect to the redox microenvironment, and NO production (Hoos et al., 2014). Mouse models on this genetic background expressing APP mutations present multiple AD like phenotypes (Wilcock et al., 2008; Colton et al., 2014; Kan et al., 2015; Badea et al., 2016). Here we assessed the differential effects of the interaction of the humanized NOS background with APOE3 and APOE4 alleles.

Our current study cannot rule out developmental effects in our mice, however, human studies point to APOE4 associated differences in asymptomatic and young carriers (Reiter et al., 2012; Piers, 2018), which may change in time (Koelewijn et al., 2019). Further studies should explore in more detail the relation between behavioral, imaging, and connectome markers.

Our findings parallel other investigations in the study of connectivity alterations associated with APOE status in human carriers and mouse models (Heise et al., 2014; Wiesmann et al., 2016; Luo et al., 2017; Korthauer et al., 2018). These studies support the presence of alterations in both functional and structural connectomes, and report separately such biomarkers. They generally point to a role for the hippocampus and its connection, and vascular function through perfusion changes, which changes may affect cognition. The importance of multimodal approaches (Wiesmann et al., 2016) and developing a framework for integrating such biomarkers has long been recognized (Madden et al., 2009), and connectomes present such an opportunity.

We argue that unique entries in a connectome contain rich information which can be further exploited at finer scales, and perhaps using different modalities. In our analyses we found significant differences in the size based ranking of the subbundles, indicating different wiring patterns in mice with different APOE alleles, and perhaps compensatory mechanisms – which are not evident at the level of whole bundle/pairwise connectivity analysis. The high resolution imaging allowed us to infer subdivisions of the bundles, based on spatial geometric relationships, and these remain to be validated using complementary methods. APOE4HN mice had consistently lower FA along the cerebellar connections, while the patterns for the interhemispheric hippocampal-cerebellar and hippocampal-piriform connections varied by subbundles, and position along the bundle, with lower FA for the largest subbundle in APOE4 carriers, but higher FAs were also observed. We observed frequently higher variability in APOE4HN mice, and in APOE4HN females compared to males. An examination of sex based differences in the hippocampal cerebellar connections indicated more consistency between the males of APOE4HN and APOE3HN genotypes, with females showing more differences with genotype in the FA curve shape (Figure 9E), and females of the same genotype showing more variability (Figures 9E–G). We note that the connections we analyzed run also through gray matter, rather than just white matter. Thus the associated FA values may be affected by aging and pathology, which led to increased FA values in gray matter.

We identified changes in volume and FA in areas which have been associated with amyloid deposition in AD patients, such as the entorhinal cortex, hippocampus, cingulate cortex and amygdala. However, our animal models do not have APP mutations predisposing them to abundant amyloid deposition, which suggests that the regions we have identified may be part of a vulnerable brain network prone to the development, propagation and deposition of misfolded proteins, proteinopathies, or involved in other pathological processes as well. While some of the significant differences in the connectome identified decreased FA along the tracts connecting these regions, the reverse was also noted. Such findings have also been reported in human APOE4 carriers, particularly at younger ages, and the effects are not uniform throughout the brain. We believe that FA may show different patterns, not only between genotypes or sexes, but even along bundles and these differences can be due to changes in the local brain microenvironment, toxicity, or myelination. Compensatory mechanisms can also play a role. In Figure 6 we note that the subbundle 5 passes largely through gray matter, so we may observe changes due to gliosis in the vicinity of such bundles.

We also found changes in the striatum gigantocellular reticular nuclei, cerebellum and cortical motor related regions. These results support the role of APOE4 (Serrano-Pozo et al., 2011) as a risk factor for Parkinson’s disease (Pankratz et al., 2006), where alpha synuclein may also be preferentially deposited in the CA2–CA3 regions of the hippocampus, insula, amygdala and cingulate cortex (Harding and Halliday, 2001; Bertrand et al., 2004). This points to shared mechanisms and vulnerable networks across neurodegenerative conditions such as AD and PD. Approximately 25% of AD patients develop PD, and 50% of PD patients develop AD after 65 years of age (Hansen et al., 1990). Moreover, 70% of LOAD patients display α-synuclein-positive LB-like inclusions in the amygdala and limbic structures (Trojanowski et al., 1998; Hamilton, 2000). Identifying differences between these vulnerable networks, based on multivariate biomarkers may help stratify patients, as e.g., dementia with Lewy bodies can be distinguished from Parkinson’s disease dementia based on the presence of Aβ deposits in the striatum (Duda et al., 2002) and hippocampus (Masliah et al., 1993).

We have shown that behavioral and imaging markers corroborate to help identify vulnerable networks in novel mouse models of pathological aging, relying on the genetic risk factor conferred by APOE4 alleles. We have also tried to gain insight into the rich information behind one single entry in a connectome. Imaging and DWI based connectomics provided multiple sensitive biomarkers to monitor the integrity of these networks or their failure in aging and disease. We hope that future work will address the mechanism underlying the switch from normal to pathological aging, and will help monitor the effects of interventions.

## Data Availability Statement

All datasets generated for this study are included in the article/supplementary material.

## Ethics Statement

The animal study was reviewed and approved by Duke IACUC.

## Author Contributions

AB, WW, CC, and DD designed the research. AB, WW, MW, RA, YQ, JS, and JW performed the research and analyzed the data. AB, WW, SK, and EG contributed new analytical tools. GJ founding director of CIVM, helped build and maintain the imaging resources at CIVM. AB, CC, WW, JS, and DD wrote the manuscript.

## Conflict of Interest

The authors declare that the research was conducted in the absence of any commercial or financial relationships that could be construed as a potential conflict of interest.

## Footnotes

1 https://www.jmp.com

2 https://github.com/portokalh/wuconnectomes

3 www.r-project.org
